# Establishment and Validation of Nomogram Model Based on Neutrophil Lymphocyte Ratio for Prognosis of Patients With Congestive Heart Failure

**DOI:** 10.1155/crp/8161090

**Published:** 2026-01-12

**Authors:** Fachao Shi, Long Wang, Enyang Wang, Caoyang Fang

**Affiliations:** ^1^ Department of Cardiology, Maanshan People’s Hospital, Maanshan, Anhui, 243000, China; ^2^ Department of Cardiology, The Second People’s Hospital of Hefei, Hefei Hospital Affiliated to Anhui Medical University, Hefei, Anhui, 230000, China, ahmu.edu.cn; ^3^ Department of Emergency, First Affiliated Hospital of University of Science and Technology of China, Anhui Provincial Hospital, Hefei, Anhui, 230000, China, ahmu.edu.cn

**Keywords:** CHF, congestive heart failure, NHANES, NLR, nomogram, prognosis

## Abstract

**Objective:**

Based on the NLR, we aim to investigate the prognostic factors of CHF and establish a nomogram model to predict the OS of CHF patients.

**Methods:**

We selected 566 CHF patients from the NHANES database surveyed between 1999 and 2018 as the study population and randomly divided the data into training and validation sets in a 7:3 ratio. We used multivariate Cox regression analysis to determine the factors affecting the prognosis of CHF patients. Additionally, we evaluated the stratification of the NLR and the nomogram total risk score using the Kaplan–Meier survival curves and log‐rank tests. To evaluate the predictive accuracy of the nomogram, we used the area under the ROC and the calibration curve method. Finally, we used decision curve analysis to explore the value of the nomogram in clinical applications.

**Results:**

Multivariate Cox regression analysis revealed that the NLR, age, and gender were risk factors affecting the OS of CHF patients, whereas hemoglobin and platelets were protective factors. We established a nomogram based on NLR, age, gender, hemoglobin, and platelets and calculated the area under the survival rate curve for 3, 5, and 10 years in both the training and validation sets, indicating good predictive capacity of the model (training set AUCs were 0.822, 0.82, and 0.803, respectively; validation set AUCs were 0.726, 0.769, and 0.775, respectively). Calibration curves and decision curve analysis indicated the model’s accuracy and clinical applicability. The risk stratification was performed using NLR and the nomogram total score, and the Kaplan–Meier survival curves and log‐rank tests showed that CHF patients with higher NLR had worse prognosis and those with lower nomogram total score had better prognosis than those in high‐risk groups. There was a significant difference in OS between the high‐ and low‐risk groups (*P* < 0.001).

**Conclusion:**

This study found that NLR, age, gender, hemoglobin, and platelets are closely related to the prognosis of CHF patients. We successfully constructed a nomogram model based on these factors, which can accurately predict the prognosis of CHF patients

## 1. Introduction

CHF represents the terminal progression of diverse cardiac conditions and remains a leading mortality cause among patients [1]. Research indicates that as CHF patients′ cardiac function classification elevates, cardiac output progressively diminishes, accompanied by a heightened risk for major adverse cardiovascular events [2]. Multiple factors including myocardial remodeling, abnormal hemodynamics, and systemic inflammation may contribute to worsening heart failure.

Despite numerous prognostic models for CHF patients including those based on the NYHA functional classification, left ventricular ejection fraction, serum sodium, and natriuretic peptides (BNP/NT‐proBNP), significant limitations persist [3–6]. The NYHA classification suffers from subjectivity, influenced by patient perception and physician assessment. While LVEF provides prognostic information, its predictive capability remains restricted in heart failure with preserved ejection fraction. Furthermore, biomarkers like NT‐proBNP lack specificity due to confounding by age, kidney function, infections, and cerebrovascular disease. Notably, existing models frequently overlook inflammation’s crucial role in CHF pathophysiology, despite its significant contribution to disease development.

Inflammatory processes are intricately linked to heart failure pathogenesis. Prospective research demonstrates significantly elevated tumor necrosis factor levels in HF patients, highlighting inflammation’s role [7]. Growing evidence confirms inflammatory system activation as crucial in HF progression [8]. Multiple studies [9–11] reveal connections between inflammatory cells and cytokines with myocardial injury, repair, and remodeling. In HF, damaged myocardium triggers cascade reactions activating inflammation. These inflammatory responses diminish myocardial contractility by affecting myocardial cells and fibroblasts. Excessive inflammatory mediators disrupt intracellular calcium transport and beta‐receptor signaling, inducing apoptosis and reducing contractility. Furthermore, inflammatory factors promote cardiac remodeling gene activation [12]. Beyond direct cardiac effects, inflammation can activate neuroendocrine systems, exacerbating cardiac remodeling.

Inflammation in HF correlates with neutrophil and lymphocyte abnormalities [13]. The NLR, an economical and reproducible inflammatory marker, offers valuable diagnostic and prognostic insights across cardiovascular conditions including coronary disease, hypertension, arrhythmias, and valvular disorders [14]. NLR integrates two immune responses: systemic inflammation (neutrophils) and physiological stress (lymphocytes) [15]. Research indicates that higher neutrophil counts in CHF patients correlate with increased inflammation, myocardial damage, worse ventricular function, and poorer outcomes [16]. Conversely, lower lymphocyte counts indicate greater physiological stress and myocardial oxygen consumption, increasing heart failure risk.

This study utilizes NHANES data to develop a nomogram incorporating NLR and other independent prognostic factors identified through multivariate Cox regression including age, gender, hemoglobin, and platelets. This model aims to address existing model limitations, comprehensively assess CHF prognosis, and provide clinicians with improved risk stratification tools for personalized treatment decisions. Incorporating the accessible, economical NLR marker into prognostic models should enhance risk identification and ultimately improve clinical outcomes.

## 2. Methods

### 2.1. Study Population

**Figure 1:**
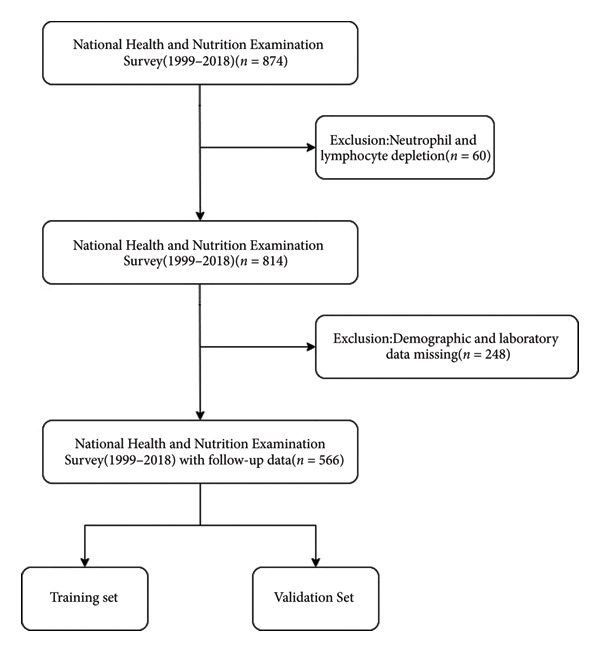
Study flowchart.

This cross‐sectional study utilized the NHANES database, screening 874 CHF patients aged over 18 years from 1999 to 2018. After excluding 60 patients lacking neutrophil and lymphocyte data and performing data cleaning, 566 CHF patients were ultimately included. These subjects were randomly allocated into training and validation cohorts at a 7:3 ratio, as shown in Figure 1. CHF diagnosis relied on the MCQ questionnaire response to “Has anyone told you that you have congestive heart failure?” with affirmative answers indicating CHF. The NHANES database is publicly accessible and received approval from the National Center for Health Statistics ethics review committee. All participants provided written informed consent before volunteering for examinations and questionnaires [17].

### 2.2. Death Information of Study Subjects

Mortality data were acquired from the National Death Index database (https://www.cdc.gov/nchs/data-link/mortality-public.htm). Individual follow‐up periods were calculated from the participation date until either death or December 31, 2019 (the NDI database’s most recent update) [18].

### 2.3. Covariates

Considering covariates potentially affecting CHF patient survival, we analyzed age, gender, race, education, marital status, PIR, diabetes, hypertension, BMI, smoking, and alcohol consumption. Race categories included non‐Hispanic Black, non‐Hispanic White, Mexican American, and Other. Education was classified as college or above, high school equivalent, or below high school. BMI was calculated as weight (kg)/height^2^(m). Smoking status comprised never smokers (< 100 lifetime cigarettes), former smokers (> 100 cigarettes, currently nonsmoking), and current smokers (> 100 cigarettes, currently smoking) [19]. Alcohol consumption was categorized as never drinkers (< 12 lifetime drinks), former drinkers (≥ 12 drinks lifetime but none past year), light drinkers (past year: ≤ 2 drinks/day women, ≤ 1 drink/day men), moderate drinkers (past year: 1–3 drinks/day women, 2–4 drinks/day men), and heavy drinkers (past year: ≥ 4 drinks/day women, ≥ 5 drinks/day men) [20].

Hypertension was defined by self‐reported history, antihypertensive medication use, systolic BP ≥ 140 mmHg, and/or diastolic BP ≥ 90 mmHg. Diabetes criteria included fasting glucose ≥ 7.0 mmol/L, 2‐h OGTT ≥ 11.1 mmol/L, random glucose ≥ 11.1 mmol/L, HbA1c ≥ 6.5%, diabetes medication/insulin use, or physician diagnosis. Laboratory measurements included serum creatinine, BUN, UA, HbA1c, and blood cell counts. eGFR was calculated using the CKD‐EPI Scr equation (21). NLR was defined as the neutrophil count divided by lymphocyte count.

### 2.4. Statistical Analysis

The measurement data were tested for normality and were expressed as mean ± standard deviation in accordance with the normal distribution; independent sample *t*‐test was used for comparison between the two groups; median (IQR, interquartile range) was used for measurement data without normal distribution, the Mann–Whitney *U* test was used for comparison between groups; enumeration data adoption rate was used for comparison between the two groups, chi‐squared test was used for comparison between the two groups;

following the NHANES Analysis and Reporting Guidelines [22], we accounted for complex sampling design and weights, using MEC sample weight (WTMEC2YR/4 + WTMEC2YR/8) for all weighted analyses. Quantitative data were tested for normality; data with normal distribution are presented as mean ± standard deviation, and comparisons between two groups were performed using independent samples *t*‐test; quantitative data with non‐normal distribution are presented as median (IQR, interquartile range), and group comparisons were performed using the Mann–Whitney *U* test. Categorical variables were expressed as *n* (%) and analyzed via chi‐squared test.

To determine optimal cutoff values for NLR and nomogram scores, we employed the *R* “maxstat” package for maximum selection rank statistical analysis (https://CRAN.R-project.org/package=maxstat) [23]. This method iteratively tests different cutoff values, calculates corresponding log‐rank statistics, and selects the value maximizing statistical significance to distinguish survival groups. Participants were subsequently divided into high/low NLR groups and high/low nomogram score groups (Figure 2). The Kaplan–Meier survival analysis and log‐rank tests verified cutoff validity by comparing survival differences between groups. *P* < 0.05 indicated statistical significance.

Figure 2Determination of NLR and nomogram total score cutoff values. Optimal cutoff values were determined using maximum selected rank statistics, and standardized log‐rank statistics are presented in the figures. (a) Determination of the cutoff value for NLR; (b) determination of the cutoff value for the total nomogram score.

**Figure:**
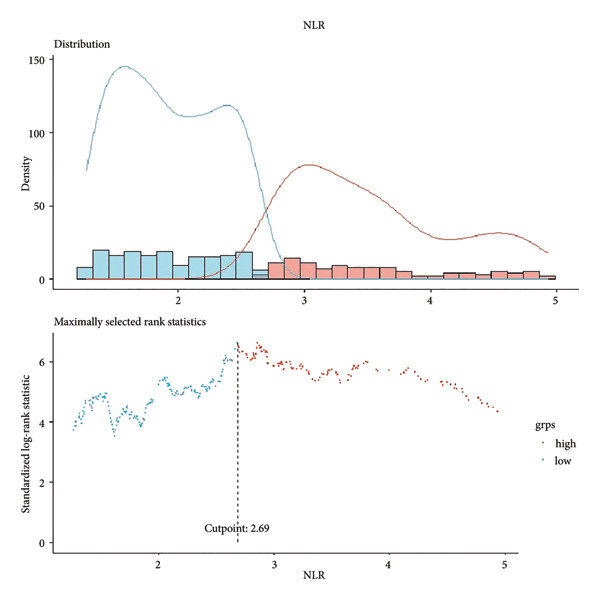
(a)

**Figure:**
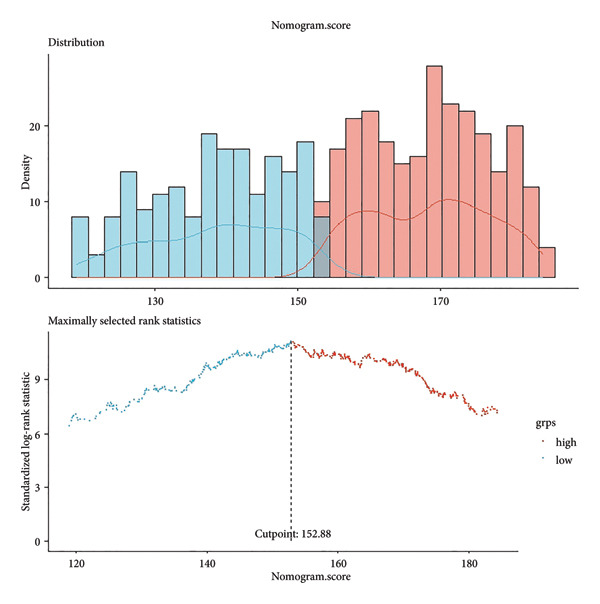
(b)

Multivariable Cox regression identified factors significantly impacting the CHF patient prognosis, enabling nomogram construction [24]. Survival outcomes for NLR and nomogram risk stratification were evaluated using the Kaplan–Meier curves and log‐rank tests. Nomogram predictive performance was assessed via area under ROC curve (AUC) and calibration curves, while decision curve analysis (DCA) explored clinical application value. All analyses were performed using *R* statistical software version 4.2.1 (http://www.r-project.org), with two‐sided *P* < 0.05 considered significant.

## 3. Results

### 3.1. Comparison of General Data Between Survival Group and Death Group

Among 566 enrolled CHF patients, 304 (53.71%) died during follow‐up. The deceased group contained more non‐Hispanic White patients, had shorter follow‐up periods, advanced age, decreased renal function, and elevated NLR levels, as detailed in Table 1.

**Table 1:** Comparison of general data between survival group and death group.

Variables	Total (*N* = 566)	Survival (*N* = 262)	Death (*N* = 304)	*Z/χ2*	*p*
Time, Median (Q1, Q3)	6.08 (3.17, 9.92)	7.12 (4.25, 11.58)	5.25 (2.31, 8.44)	6.644	< 0.001
Age, Median (Q1, Q3)	69.00 (60.00, 78.00)	63.00 (52.25, 72.00)	74.00 (65.00, 80.00)	9.312	< 0.001
PIR, Median (Q1, Q3)	1.61 (1.02, 3.00)	1.44 (0.90, 3.21)	1.67 (1.11, 2.78)	1.011	0.312
BMI, Median (Q1, Q3)	29.85 (25.91, 34.93)	30.85 (26.90, 37.27)	29.02 (25.27, 33.40)	3.688	< 0.001
eGFR, Median (Q1, Q3)	67.60 (48.78, 88.01)	78.34 (58.60, 96.59)	58.46 (40.06, 79.28)	8.216	< 0.001
NLR, Median (Q1, Q3)	2.41 (1.69, 3.40)	2.13 (1.50, 2.86)	2.68 (1.90, 3.89)	5.574	< 0.001
Hemoglobin, Median (Mean ± SD)	13.76 ± 1.78	13.87 ± 1.71	13.66 ± 1.84	1.40	0.163
Platelet, Median (Mean ± SD)	227.03 ± 76.02	229.96 ± 71.88	224.50 ± 79.45	0.85	0.395
HbA1c, Median (Mean ± SD)	6.30 ± 1.33	6.27 ± 1.37	6.32 ± 1.29	0.40	0.690
Creatinine, Median (Mean ± SD)	114.52 ± 104.48	93.75 ± 66.13	132.43 ± 126.08	4.66	< 0.001
UA, Median (Q1, Q3)	368.80 (303.30, 450.52)	365.80 (297.40, 428.30)	374.70 (313.72, 463.90)	2.853	0.004
BUN, Median (Q1, Q3)	6.07 (4.64, 8.92)	5.36 (4.02, 7.14)	7.14 (5.36, 10.35)	7.801	< 0.001
Sex, *n* (%)				3.664	0.056
Female	250 (44.2%)	127 (48.5%)	123 (40.5%)		
Male	316 (55.8%)	135 (51.5%)	181 (59.5%)		
Race, *n* (%)				15.115	0.002
Mexican American	52 (9.2%)	27 (10.3%)	25 (8.2%)		
Non‐Hispanic Black	123 (21.7%)	64 (24.4%)	59 (19.4%)		
Non‐Hispanic White	328 (58.0%)	131 (50.0%)	197 (64.8%)		
Other	63 (11.1%)	40 (15.3%)	23 (7.6%)		
Marital, *n* (%)				12.799	0.005
Divorced	80 (14.1%)	43 (16.4%)	37 (12.2%)		
Married	286 (50.5%)	142 (54.2%)	144 (47.4%)		
Never married	32 (5.7%)	18 (6.9%)	14 (4.6%)		
Other	168 (29.7%)	59 (22.5%)	109 (35.9%)		
Education, *n* (%)				4.541	0.103
High school or equivalent	131 (23.1%)	66 (25.2%)	65 (21.4%)		
Less than high school	221 (39.0%)	90 (34.4%)	131 (43.1%)		
Some college or above	214 (37.8%)	106 (40.5%)	108 (35.5%)		
Smoke, *n* (%)				3.307	0.191
Former	209 (36.9%)	90 (34.4%)	119 (39.1%)		
Never	241 (42.6%)	110 (42.0%)	131 (43.1%)		
Now	116 (20.5%)	62 (23.7%)	54 (17.8%)		
Alcohol, *n* (%)				20.391	< 0.001
Former	206 (36.4%)	73 (27.9%)	133 (43.8%)		
Heavy	52 (9.2%)	33 (12.6%)	19 (6.2%)		
Mild	181 (32.0%)	92 (35.1%)	89 (29.3%)		
Moderate	42 (7.4%)	25 (9.5%)	17 (5.6%)		
Never	85 (15.0%)	39 (14.9%)	46 (15.1%)		
Hypertension, *n* (%)				0.256	0.613
No	100 (17.7%)	44 (16.8%)	56 (18.4%)		
Yes	466 (82.3%)	218 (83.2%)	248 (81.6%)		
Diabetes, *n* (%)				4.434	0.109
Borderline	93 (16.4%)	39 (14.9%)	54 (17.8%)		
Yes	276 (48.8%)	120 (45.8%)	156 (51.3%)		
No	197 (34.8%)	103 (39.3%)	94 (30.9%)		
CHD, *n* (%)				0.492	0.483
No	339 (59.9%)	161 (61.5%)	178 (58.6%)		
Yes	227 (40.1%)	101 (38.5%)	126 (41.4%)		

*Note:* Date are presented as mean ± SD, median (Q1, Q3) or *n* (%).

Abbreviations: BMI = body mass index; BUN = blood urea nitrogen; CHD = coronary heart disease; eGFR = estimated glomerular filtration rate; HbA1c = glycosylated hemoglobin; NLR = neutrophil‐to‐lymphocyte ratio; PIR = poverty income ratio; UA = uric acid.

### 3.2. Comparison of General Data of Patients Between Training Set and Validation Set

This study included 566 CHF patients randomly allocated into training and validation sets at a 7:3 ratio. Overall mortality was 53.71% (304 patients), with 217 deaths (54.52%) in the training set and 87 deaths (51.78%) in the validation set. Statistically significant differences (*P* < 0.05) were observed between training and validation cohorts regarding hemoglobin levels and smoking history, as presented in Table 2.

**Table 2:** Comparison of general data of patients between training set and validation set.

Variables	Total (*N* = 566)	Test data (*N* = 168)	Training data (*N* = 398)	*Z/χ2*	*p*
Time, Median (Q1, Q3)	6.08 (3.17, 9.92)	5.67 (2.88, 9.75)	6.33 (3.35, 10.06)	1.054	0.292
Age, Median (Q1, Q3)	69.00 (60.00, 78.00)	68.00 (59.00, 78.25)	70.00 (60.00, 77.00)	0.091	0.927
PIR, Median (Q1, Q3)	1.61 (1.02, 3.00)	1.67 (1.07, 2.93)	1.55 (1.01, 3.00)	0.763	0.445
BMI, Median (Q1, Q3)	29.85 (25.91, 34.93)	30.48 (26.14, 35.51)	29.69 (25.85, 34.77)	1.070	0.285
eGFR, Median (Q1, Q3)	67.60 (48.78, 88.01)	67.47 (47.25, 88.08)	67.73 (50.32, 87.74)	0.040	0.968
NLR, Median (Q1, Q3)	2.41 (1.69, 3.40)	2.44 (1.73, 3.36)	2.40 (1.67, 3.43)	0.220	0.826
Hemoglobin, Median (Mean ± SD)	13.76 ± 1.78	13.70 ± 1.74	13.48 ± 1.80	−0.48	0.030
Platelet, Median (Mean ± SD)	227.03 ± 76.02	228.35 ± 83.47	226.46 ± 72.70	0.27	0.786
HbA1c, Median (Mean ± SD)	6.30 ± 1.33	6.49 ± 1.61	6.41 ± 1.18	1.99	0.548
Creatinine, Median (Mean ± SD)	114.52 ± 104.48	116.11 ± 87.43	113.85 ± 111.10	0.24	0.814
UA, Median (Q1, Q3)	368.80 (303.30, 450.52)	374.70 (309.30, 459.48)	368.80 (303.30, 446.10)	0.567	0.571
BUN, Median (Q1, Q3)	6.07 (4.64, 8.92)	6.07 (4.64, 8.93)	6.07 (4.64, 8.57)	0.072	0.943
Status, *n* (%)				0.356	0.551
Survival	262 (46.3%)	81 (48.2%)	181 (45.5%)		
Death	304 (53.7%)	87 (51.8%)	217 (54.5%)		
Sex, *n* (%)				0.022	0.883
Female	250 (44.2%)	75 (44.6%)	175 (44.0%)		
Male	316 (55.8%)	93 (55.4%)	223 (56.0%)		
Race, *n* (%)				6.478	0.091
Mexican American	52 (9.2%)	14 (8.3%)	38 (9.5%)		
Non‐Hispanic Black	123 (21.7%)	29 (17.3%)	94 (23.6%)		
Non‐Hispanic White	328 (58.0%)	99 (58.9%)	229 (57.5%)		
Other	63 (11.1%)	26 (15.5%)	37 (9.3%)		
Marital, *n* (%)				1.869	0.600
Divorced	80 (14.1%)	21 (12.5%)	59 (14.8%)		
Married	286 (50.5%)	92 (54.8%)	194 (48.7%)		
Never married	32 (5.7%)	8 (4.8%)	24 (6.0%)		
Other	168 (29.7%)	47 (28.0%)	121 (30.4%)		
Education, *n* (%)				1.097	0.578
High school or equivalent	131 (23.1%)	41 (24.4%)	90 (22.6%)		
Less than high school	221 (39.0%)	69 (41.1%)	152 (38.2%)		
Some college or above	214 (37.8%)	58 (34.5%)	156 (39.2%)		
Smoke, *n* (%)				8.501	0.014
Former	209 (36.9%)	77 (45.8%)	132 (33.2%)		
Never	241 (42.6%)	59 (35.1%)	182 (45.7%)		
Now	116 (20.5%)	32 (19.0%)	84 (21.1%)		
Alcohol, *n* (%)				1.726	0.786
Former	206 (36.4%)	57 (33.9%)	149 (37.4%)		
Heavy	52 (9.2%)	16 (9.5%)	36 (9.0%)		
Mild	181 (32.0%)	57 (33.9%)	124 (31.2%)		
Moderate	42 (7.4%)	15 (8.9%)	27 (6.8%)		
Never	85 (15.0%)	23 (13.7%)	62 (15.6%)		
Hypertension, *n* (%)				0.313	0.576
No	100 (17.7%)	32 (19.0%)	68 (17.1%)		
Yes	466 (82.3%)	136 (81.0%)	330 (82.9%)		
Diabetes, *n* (%)				3.929	0.140
Borderline	93 (16.4%)	34 (20.2%)	59 (14.8%)		
Yes	276 (48.8%)	84 (50.0%)	192 (48.2%)		
No	197 (34.8%)	50 (29.8%)	147 (36.9%)		
CHD, *n* (%)				0.199	0.655
No	339 (59.9%)	103 (61.3%)	236 (59.3%)		
Yes	227 (40.1%)	65 (38.7%)	162 (40.7%)		

*Note:* Date are presented as mean ± SD, median (Q1, Q3) or *n* (%).

Abbreviations: BMI = body mass index; BUN = blood urea nitrogen; CHD = coronary heart disease; eGFR = estimated glomerular filtration rate; HbA1c = glycosylated hemoglobin; NLR = neutrophil‐to‐lymphocyte ratio; PIR = poverty income ratio; UA = uric acid.

### 3.3. Risk Factor Analysis of Prognosis in CHF Patients

Univariate Cox regression identified multiple factors associated with CHF patient overall survival (OS): gender, age, race, smoking history, drinking history, BMI, eGFR, NLR, hemoglobin, platelets, creatinine, uric acid, and blood urea nitrogen (*P* < 0.05).

Subsequent multivariate Cox regression revealed several independent prognostic factors: NLR (HR = 1.12, 95% CI: 1.054–1.19), age (HR = 1.071, 95% CI: 1.046–1.096), and gender (HR = 2.146, 95% CI: 1.487–3.096) emerged as risk factors for reduced OS (*P* < 0.05), while hemoglobin (HR = 0.852, 95% CI: 0.748–0.909) and platelets (HR = 0.998, 95% CI: 0.996–1) functioned as protective factors (*P* < 0.05). Complete results appear in Table 3.

**Table 3:** Univariate and multivariate Cox regression analyses.

Variables	Univariate Cox regression analysis				Multivariate Cox regression analysis			
	*β*	*z*	HR (95%CI)	*p*	*β*	*z*	HR (95%CI)	*p*
Age	0.068	8.815	1.071 (1.055, 1.087)	< 0.001	0.068	5.761	1.071 (1.046, 1.096)	< 0.001
Sex								
Female			Reference				Reference	
Male	0.332	2.364	1.394 (1.058, 1.835)	0.018	0.764	4.081	2.146 (1.487, 3.096)	< 0.001
Race								
Mexican American			Reference				Reference	
Non‐Hispanic Black	0.350	1.247	1.419 (0.819, 2.461)	0.212	0.190	0.627	1.209 (0.668, 2.190)	0.530
Non‐Hispanic White	0.605	2.402	1.831 (1.118, 3.001)	0.016	0.063	0.236	1.064 (0.634, 1.787)	0.813
Other	0.273	0.776	1.313 (0.660, 2.613)	0.438	0.031	0.087	1.032 (0.507, 2.098)	0.931
Marital								
Divorced			Reference					
Married	−0.170	0.791	0.844 (0.554, 1.285)	0.429				
Never married	−0.460	1.199	0.631 (0.298, 1.339)	0.230				
Other	0.340	1.544	1.406 (0.912, 2.165)	0.123				
PIR	−0.005	0.108	0.995 (0.907, 1.091)	0.914				
Education								
High school or equivalent			Reference					
Less than high school	0.006	0.030	1.006 (0.702, 1.441)	0.976				
Some college or above	−0.036	0.190	0.965 (0.668, 1.394)	0.849				
Smoke								
Former			Reference				Reference	
Never	0.040	0.264	1.041 (0.774, 1.398)	0.792	0.335	1.931	1.398 (0.995, 1.963)	0.053
Now	−0.460	2.243	0.631 (0.422, 0.944)	0.025	0.311	1.342	1.365 (0.866, 2.150)	0.180
Alcohol								
Former			Reference				Reference	
Heavy	−0.644	2.250	0.525 (0.300, 0.920)	0.024	0.625	1.950	1.869 (0.997, 3.504)	0.051
Mild	−0.265	1.621	0.767 (0.557, 1.057)	0.105	−0.131	0.770	0.877 (0.628, 1.224)	0.441
Moderate	−0.480	1.565	0.619 (0.340, 1.129)	0.118	0.178	0.552	1.195 (0.635, 2.249)	0.581
Never	−0.130	0.646	0.878 (0.591, 1.304)	0.518	−0.362	1.512	0.697 (0.436, 1.113)	0.131
Hypertension								
No			Reference					
Yes	0.308	1.707	1.360 (0.955, 1.937)	0.088				
Diabetes								
Borderline			Reference					
Yes	0.295	1.437	1.344 (0.898, 2.010)	0.151				
No	0.006	0.027	1.006 (0.658, 1.538)	0.978				
BMI	−0.026	2.546	0.975 (0.956, 0.994)	0.011	−0.004	0.318	0.996 (0.972, 1.021)	0.750
eGFR	−0.022	8.817	0.978 (0.973, 0.983)	< 0.001	−0.006	0.893	0.994 (0.982, 1.007)	0.372
NLR	0.157	6.762	1.170 (1.118, 1.224)	< 0.001	0.113	3.662	1.120 (1.054, 1.190)	< 0.001
Hemoglobin	−0.128	3.229	0.879 (0.814, 0.951)	0.001	−0.193	3.859	0.825 (0.748, 0.909)	< 0.001
Platelet	−0.004	3.647	0.996 (0.994, 0.998)	< 0.001	−0.002	2.200	0.998 (0.996, 1.000)	0.028
HbA1c	0.083	1.661	1.086 (0.985, 1.198)	0.097				
Creatinine	0.001	3.288	1.001 (1.001, 1.002)	0.001	0.002	1.658	1.002 (1.000, 1.003)	0.097
UA	0.003	4.388	1.003 (1.002, 1.004)	< 0.001	0.001	0.811	1.001 (0.999, 1.002)	0.417
BUN	0.106	8.149	1.111 (1.084, 1.140)	< 0.001	0.015	0.637	1.015 (0.969, 1.063)	0.524
CHD								
No			Reference					
Yes	0.154	1.120	1.167 (0.891, 1.527)	0.263				

*Note:* Date are presented as median (Q1, Q3) or *n* (%).

Abbreviations: BMI = body mass index; BUN = blood urea nitrogen; CHD = coronary heart disease; eGFR = estimated glomerular filtration rate; HbA1c = glycosylated hemoglobin; NLR = neutrophil‐to‐lymphocyte ratio; PIR = poverty income ratio; UA = uric acid.

### 3.4. Development and Validation of Nomogram Model

**Figure 3:**
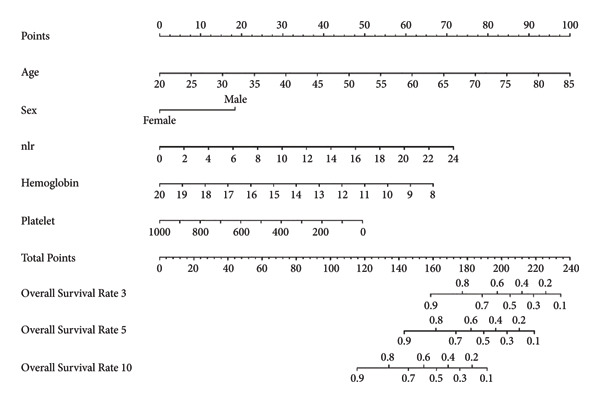
NLR‐based nomogram model for survival prediction in CHF patients. Method of application: First, determine the patient’s age, gender, NLR value, hemoglobin, and platelet count. Find the scale corresponding to each variable on the nomogram and determine a value on the scale according to the actual situation of the patient. The values of all variables were summed to obtain the total points. Finally, the corresponding survival prediction value of patients can be obtained by finding the corresponding position on the overall score axis and projecting downward to the 3‐, 5‐, and 10‐year survival axes.

Based on multivariate Cox regression, variables with *P* < 0.05 (NLR, gender, age, hemoglobin, and platelets) were incorporated into the nomogram displayed in Figure 3. ROC curve analysis demonstrated AUC values for 3‐year, 5‐year, and 10‐year survival predictions of 0.822, 0.82, and 0.803 in the training set and 0.726, 0.769, and 0.775 in the validation set, respectively (Figure 4). The nomogram effectively predicted OS probability at these time points, with calibration curves confirming strong agreement between predicted and actual outcomes (Figure 5). DCA validated the nomogram’s clinical utility for predicting OS across all timeframes (Figure 6). Maximum selection rank statistical analysis established an optimal NLR cutoff value of 2.41 (Figure 2(a)) and an optimal nomogram score cutoff value of X (to be completed with actual results) (Figure 2(b)). These thresholds enabled patient stratification into high‐/low‐risk groups. The Kaplan–Meier analysis revealed significantly lower survival rates in the high NLR group compared to those in the low NLR group (*P* < 0.001, Figure 7(a)), with similar findings between high and low nomogram score groups (*P* < 0.001, Figure 7(b)). These results confirm both NLR and nomogram scores as effective tools for CHF patient risk stratification.

Figure 4ROC curves for nomogram models predicting 3‐year, 5‐year, and 10‐year OS in CHF patients. (a) Training set; (b) validation set.

**Figure:**
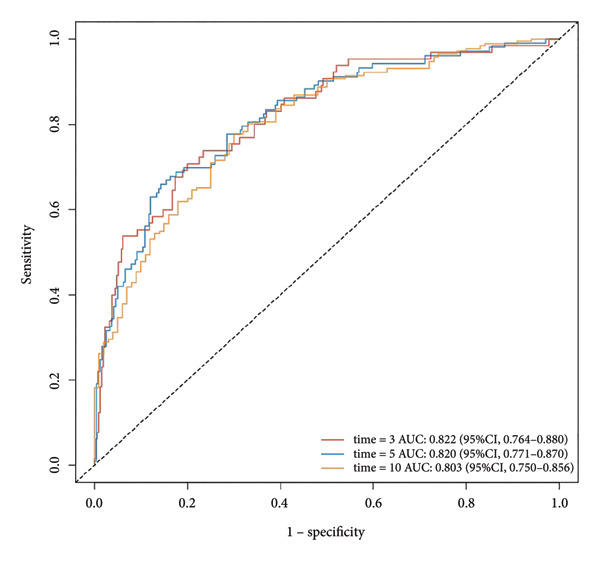
(a)

**Figure:**
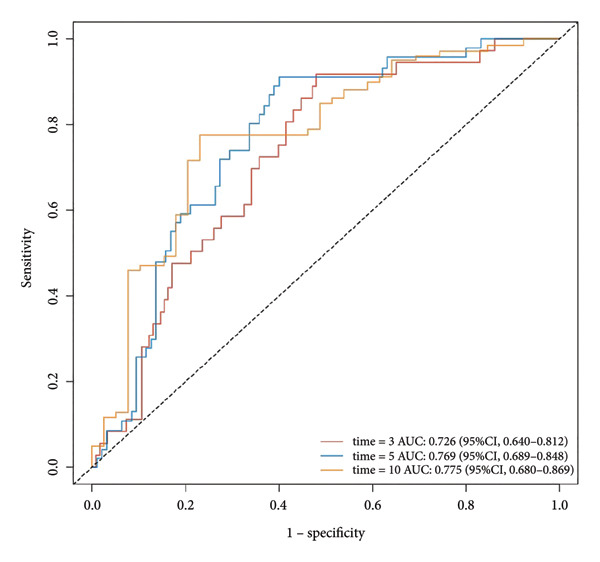
(b)

Figure 5Calibration curve of nomogram model for predicting 3‐year, 5‐year, and 10‐year OS in CHF patients. (a) Training set; (b) validation set.

**Figure:**
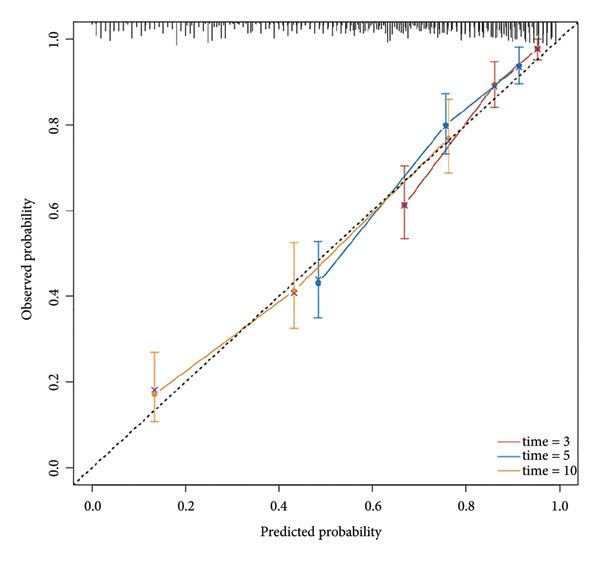
(a)

**Figure:**
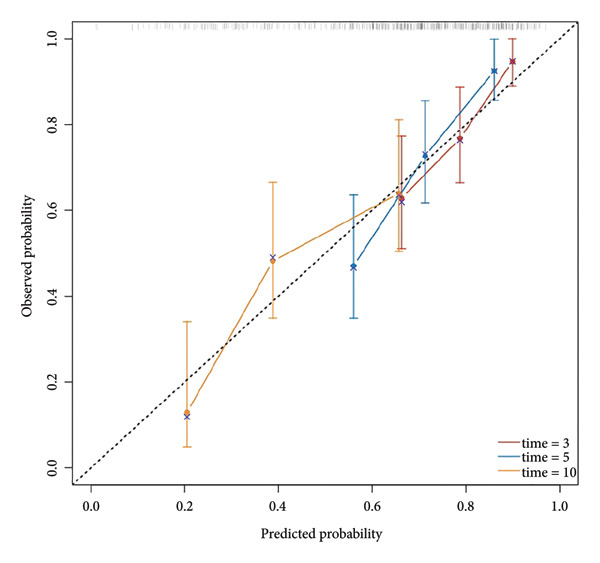
(b)

Figure 6Decision curve analysis of nomogram models predicting 3‐, 5‐, and 10‐year overall survival in CHF patients. (a) Training set; (b) validation set. The decision curve shows the net benefit that can be obtained by using this nomogram model for prediction at different threshold probabilities. The higher the curve, the greater the value of clinical application.

**Figure:**
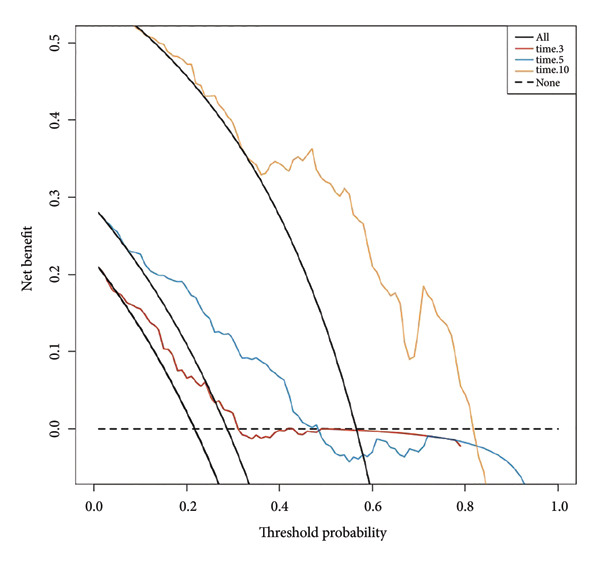
(a)

**Figure:**
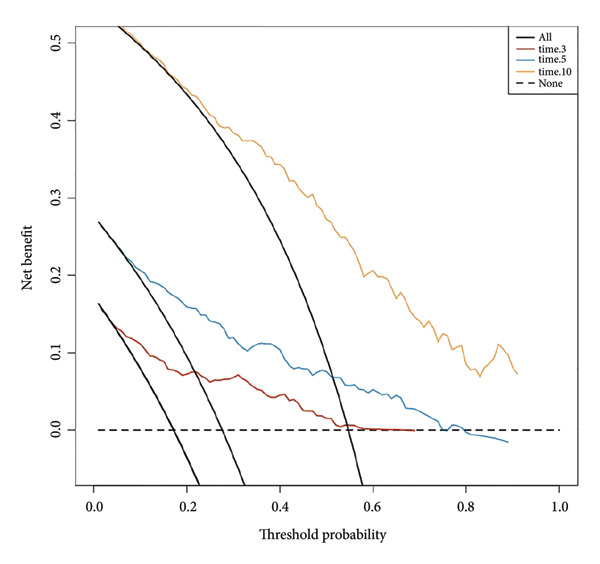
(b)

Figure 7Kaplan–Meier survival curves for NLR strata (a) and nomogram total score strata (b) in CHF patients. Log‐rank test was used to compare survival differences between different risk groups. *P* < 0.001 indicates a statistically significant difference in survival between the high‐risk and low‐risk groups.

**Figure:**
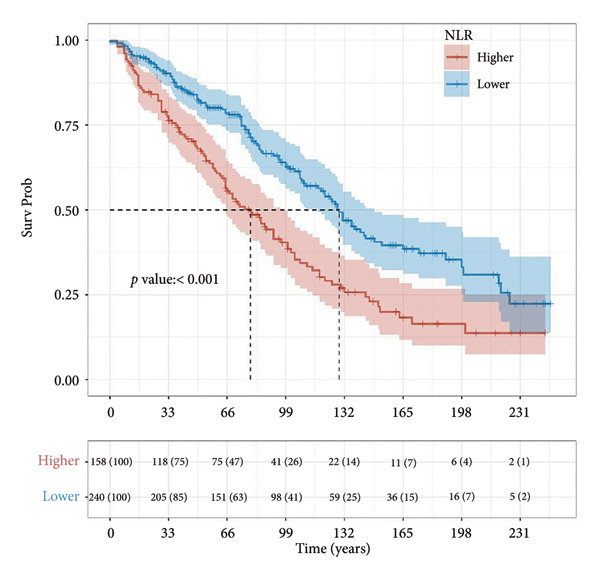
(a)

**Figure:**
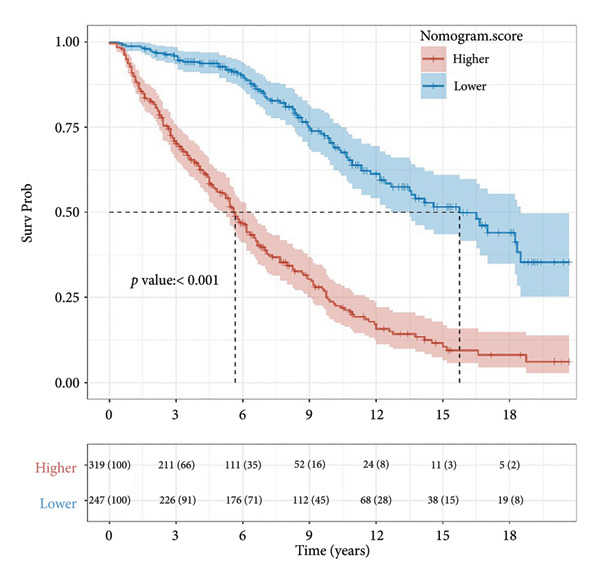
(b)

## 4. Discussion

Heart failure represents a clinical syndrome with multiple causative mechanisms leading to cardiac structural and functional abnormalities. Systemic immune activation and inflammatory processes contribute significantly to its pathophysiology [24]. The relationship between inflammation and heart failure was first documented by Levine et al. [25] in 1990, who observed tumor necrosis factor levels in HFrEF patients. White blood cell subtypes serve as classical inflammatory markers, with research indicating that elevated neutrophil counts correlate with increased heart failure incidence [26], while reduced lymphocyte counts associate with poorer cardiac function and survival rates [27]. The neutrophil‐to‐lymphocyte ratio (NLR) has emerged as a novel inflammatory indicator [28]. Curran et al. [29] demonstrated that NLR exhibits a superior predictive value compared to individual neutrophil or lymphocyte counts.

Numerous studies have established NLR’s prognostic significance in heart failure. Research on acute decompensated heart failure (ADHF) patients [30] revealed NLR’s predictive capacity for long‐term mortality and 30‐day readmission risk, with higher NLR correlating with worse outcomes. Another investigation [31] found significantly elevated NLR in heart failure patients versus controls, with multivariate logistic regression confirming NLR as an independent mortality predictor. Multiple studies support NLR’s role as an independent predictor of adverse outcomes in heart failure [32]. In cardiac resynchronization therapy research [33], nonresponsive patients exhibited significantly higher NLR than responsive patients. Multivariate analysis identified NLR as an independent predictor of therapy nonresponsiveness, with NLR > 3.45 increasing the nonresponse risk by 12.2‐fold. Additional research [34] demonstrated NLR’s ability to predict adverse outcomes after left ventricular assist device implantation, including all‐cause mortality and right ventricular failure. Furthermore, increased NLR in end‐stage heart failure patients correlates with elevated heart transplantation risk and independently predicts in‐hospital and 1‐year posttransplant mortality [35].

Our study identified non‐Hispanic White CHF patients as the predominant mortality group, characterized by shorter follow‐up periods, advanced age, compromised renal function, and elevated NLR. Multivariate Cox regression confirmed NLR as an independent risk factor, with each unit increase raising mortality risk by 12%. The Kaplan–Meier analysis demonstrated an inverse relationship between NLR levels and cumulative survival rates. These findings align with previous research [15, 36] establishing NLR as an independent predictor of rehospitalization, in‐hospital mortality, and adverse outcomes in both acute and chronic heart failures. Curran et al. [29] further demonstrated NLR’s prognostic value across HFrEF and HFpEF subgroups. Based on multivariate analysis, we developed a nomogram predicting OS in CHF patients. The ROC curve evaluation confirmed high predictive accuracy for 3‐year, 5‐year, and 10‐year survival in both training and validation cohorts. Risk stratification using NLR values and nomogram scores revealed significant survival differences between high‐ and low‐risk groups, with Kaplan–Meier curves and log‐rank tests confirming worse outcomes in patients with elevated NLR and higher nomogram scores.

Extensive literature supports NLR’s utility in cardiovascular disease diagnosis and prognosis, reflecting heart failure severity with strong predictive capabilities. The pathophysiological basis involves activated neutrophils releasing myeloperoxidase, acid phosphatase, and elastase, promoting tissue damage and cardiac dysfunction, resulting in higher NYHA classification and reduced LVEF. Simultaneously, lymphocyte reduction linked to impaired proliferation, neutrophil activation, and lymphocyte apoptosis from elevated cortisol and catecholamines contributes to cardiac deterioration [37, 38]. While atrial natriuretic peptide and NT‐proBNP remain established heart failure markers [27], NT‐proBNP levels fluctuate with infection, renal impairment, and cerebrovascular disease. NLR offers advantages through accessibility, affordability, and measurement stability. Although the NLR‐HFpEF relationship requires further validation, growing HFpEF prevalence and emerging understanding of inflammatory mechanisms suggest continued research into NLR’s prognostic value in this population. NLR’s routine availability and low cost enhance its potential for widespread clinical implementation.

Chronic obstructive pulmonary disease represents a significant comorbidity in CHF patients, with their coexistence substantially increasing mortality risk [39]. COPD negatively impacts CHF through multiple mechanisms: chronic airway obstruction and emphysema elevate pulmonary arterial pressure and right ventricular burden, compromising overall cardiac function; the persistent inflammatory state in COPD amplifies systemic inflammatory responses, exacerbating myocardial damage and ventricular remodeling; additionally, COPD‐related dyspnea limits physical activity, reducing quality of life and potentially compromising CHF treatment adherence.

Our study has several limitations: First, we relied solely on database information for model construction and validation, necessitating further multicenter prospective studies to confirm its accuracy. Second, our cohort comprised exclusively American CHF patients, requiring additional research to establish applicability to other populations. Third, we were unable to incorporate COPD into our nomogram model, representing a significant limitation. Future research should include COPD as a key covariate or validate our nomogram’s predictive capacity in combined COPD–CHF populations to enhance risk assessment precision. We suggest that subsequent studies collect more comprehensive clinical data, including COPD diagnosis, severity classification, and treatment approaches, to better evaluate its impact on CHF outcomes.

## 5. Conclusions

Our newly developed nomogram effectively predicts 3‐, 5‐, and 10‐year survival probabilities in CHF patients. This prognostic tool equips clinicians and patients with valuable information for creating individualized treatment plans, ultimately enhancing patient quality of life.

**Nomenclature**
NLR: Neutrophil–lymphocyte ratio
CHF: Congestive heart failure
OS: Overall survival
ROC: Receiver operating characteristic curve
AUC: Area under curve
PIR: Poverty income ratio
BMI: Body mass index
HbA1c: Glycosylated hemoglobin
UA: Uric acid
BUN: Blood urea nitrogen
eGFR: Estimated glomerular filtration rate
CHD: Coronary heart disease

## Ethics Statement

The study was conducted according to the Declaration of Helsinki. All information from the NHANES program is freely available to the public and therefore does not require approval from the Medical Ethics Committee.

## Disclosure

All authors approved the submitted version.

## Conflicts of Interest

The authors declare no conflicts of interest.

## Author Contributions

Fachao Shi wrote the main manuscript text. Long Wang, Enyang Wang, and Caoyang Fang prepared Tables 1–3 and Figures 1–7. Fachao Shi contributed to the framework and review of the manuscript accordingly. All authors reviewed the manuscript. Fachao Shi and Long Wang contributed equally to the article.

## Funding

No funding was received for this manuscript.

## Data Availability

The data that support the findings of this study are available from the corresponding author upon reasonable request.
